# Comparison of Two Alternative Procedures to Obtain Packed Red Blood Cells for β-Thalassemia Major Transfusion Therapy

**DOI:** 10.3390/biom11111638

**Published:** 2021-11-04

**Authors:** Davide Schiroli, Lucia Merolle, Eleonora Quartieri, Roberta Chicchi, Tommaso Fasano, Tiziana De Luca, Giuseppe Molinari, Stefano Pulcini, Thelma A. Pertinhez, Erminia Di Bartolomeo, Rino Biguzzi, Roberto Baricchi, Chiara Marraccini

**Affiliations:** 1 Transfusion Medicine Unit, Azienda USL-IRCCS di Reggio Emilia, 42123 Reggio Emilia, Italy; davide.schiroli@ausl.re.it (D.S.); Eleonora.Quartieri@ausl.re.it (E.Q.); tiziana.deluca@ausl.re.it (T.D.L.); giusemoli@gmail.com (G.M.); stefanopulcini1993@gmail.com (S.P.); erminia.dibartolomeo@ausl.re.it (E.D.B.); roberto.baricchi@ausl.re.it (R.B.); chiara.marraccini@ausl.re.it (C.M.); 2 Department of Medicine and Surgery, University of Parma, 43126 Parma, Italy; thelma.pertinhez@unipr.it; 3 Immunohematology and Transfusion Medicine Unit, Romagna Hub Laboratory, 47522 Pievesestina, Italy; roberta.chicchi@auslromagna.it (R.C.); rino.biguzzi@auslromagna.it (R.B.); 4 Clinical Chemistry and Endocrinology Laboratory, Azienda USL-IRCCS di Reggio Emilia, 42123 Reggio Emilia, Italy; tommaso.fasano@ausl.re.it

**Keywords:** β-thalassemia, packed red blood cells, metabolites, iron overload, blood transfusion

## Abstract

β-thalassemia major (βTM) patients require frequent blood transfusions, with consequences that span from allogenic reactions to iron overload. To minimize these effects, βTM patients periodically receive leucodepleted packed red blood cells (P-RBCs) stored for maximum 14 days. The aim of this study was to compare two alternative routine procedures to prepare the optimal P-RBCs product, in order to identify differences in their content that may somehow affect patients’ health and quality of life (QoL). In method 1, blood was leucodepleted and then separated to obtain P-RBCs, while in method 2 blood was separated and leucodepleted after removal of plasma and buffycoat. Forty blood donors were enrolled in two independent centers; couples of phenotypically matched whole blood units were pooled, divided in two identical bags and processed in parallel following the two methods. Biochemical properties, electrolytes and metabolic composition were tested after 2, 7 and 14 days of storage. Units prepared with both methods were confirmed to have all the requirements necessary for βTM transfusion therapy. Nevertheless, RBCs count and Hb content were found to be higher in method-1, while P-RBCs obtained with method 2 contained less K^+^, iron and storage lesions markers. Based on these results, both methods should be tested in a clinical perspective study to determine a possible reduction of transfusion-related complications, improving the QoL of βTM patients, which often need transfusions for the entire lifespan.

## 1. Introduction

The defective synthesis of the β-globin chain of hemoglobin (Hb) is the cause of a group of hereditary blood diseases named β-thalassemias. In the case of β-thalassemia major (βTM) the absence of β-globin chains induces massive red blood cells (RBCs) hemolysis that leads to a severe pathological condition and eventually to death, if untreated, during childhood [1].

Up to the present day, the only curative therapy for βTM was the hematopoietic stem cell transplantation, which is available only in a limited set of patients, due to the age of the recipient and the lack of matching donors [1]. Recently, gene therapy and editing approaches, as well as a new generation of experimental drugs, hold the promise for a definitive treatment for βTM [1,2,3,4,5]. In the meantime, transfusion with packed RBCs (P-RBCs) remains the main therapeutic option for βTM patients.

P-RBCs transfusion is able to correct anemia, block erythropoiesis, counteract growth impairment, prevent bone deformities and inhibit iron absorption in the gastrointestinal tract caused by the elevated levels of ineffective erythropoiesis [2]. For βTM patients, transfusions are necessary for the entire lifespan, at regular intervals (every two-three weeks) [1,2].

Nevertheless, frequent transfusions lead to a series of complications that significantly impact on patients’ quality of life (QoL) [1,2]. All transfusion-dependent thalassemics accumulate iron in organs and tissues, which increases the risk of endocrine complications, liver fibrosis and heart disfunction, the latter being the leading cause of death (71% of cases). Myocardial iron deposition triggers the development of heart diseases [6], such as heart failure and arrhythmias, which are undoubtedly the most important complications of iron overload in βTM patients. Iron also accumulates in the endocrine glands causing hypothyroidism, hypoparathyroidism, adrenal insufficiency, diabetes mellitus, and hypogonadism [7]. Transfusion-dependent iron overload can also be responsible for cirrhosis [1]. Iron chelation therapy is thus essential: its usage has been implemented in all transfused βTM patients, and the recent development of more efficient chelators further contributed to increase life expectancy [1,2,7]. Both transfusion therapy and the treatment of its adverse effects represent an important cost and burden for the healthcare systems, particularly in Southern Europe where βTM is prevalent (in Italy there were 2981 registered individuals with βTM in 2019 [8]). Considering these complications and the desired therapeutic effect, the guidelines for the transfusion therapy in βTM define that P-RBCs, which are leucodepleted to reduce the incidence of undesired immune response, should also contain the highest concentration possible of Hb (at least 40 g/unit) [2,9].

Identifying the most efficient method to obtain P-RBCs with the highest content of Hb and the best safety features (minimum amount of haemolysis, storage lesions [10,11,12,13] and pro-inflammatory factors [14,15]) would significantly improve βTM patients’ QoL, reducing both the transfusion frequency and the risk of adverse effects. Clinical trials on βTM patients mostly focus on the efficacy of iron chelating agents, while almost no attention is dedicated to the transfusion therapy itself: to our knowledge, only a recent randomized crossover clinical trial tried to compare βTM patients treated with P-RBCs obtained with two alternative methods routinely used in the clinical practice to prepare leucodepleted RBCs [16]. In this study, the transfusion interval was improved after treating with P-RBCs with the highest Hb content. However, the study was conducted only over 1-year period and did not take into consideration neither long-term adverse effects nor iron overload, which is the most serious complication of transfusion dependency.

Here we focused on the overall quality and characteristics of two routinely used P-RBCs, in order to identify differences in the alternative products that may have some long-term detrimental effects on patients. In the first method, which was proved to increase the transfusion interval [16], whole blood is leucodepleted and separated afterwards to remove plasma. In the second method, plasma and buffy coat are first removed and the remaining RBCs are then leucodepleted [8,17]. Through a multi-center study involving 40 blood donors, we investigated how the two procedures affect the features of P-RBCs destined to βTM patients, assessing hematologic parameters, hemolysis, major metabolites content, iron metabolism, electrolytes and cytokines concentrations.

## 2. Materials and Methods

### 2.1. Ethics

This multi-center study involved the Transfusion Medicine Unit of the AUSL-IRCCS di Reggio Emilia and the Immunohematology and Transfusion Medicine Unit of the Romagna Hub Laboratory (Pievesestina, Italy). The protocol was approved by the Reggio Emilia Ethical Committee on 24 March 2020 (protocol number 2020/00038348) and by the Romagna Ethical Committee on 30 July 2020 (protocol number 6339/2020). All volunteer donors who participated in this study signed an informed consent according to the Declaration of Helsinki. Donors were recruited at both centers, while all the experiments were performed at the Transfusion Medicine Unit of Reggio Emilia.

### 2.2. Blood Collection

Blood donors were 25–50 year old males. Forty whole blood units (450 mL ± 45 mL) were collected at the Transfusion Medicine Unit of the AUSL-IRCCS (20 units) and at the Transfusion Medicine Unit of the Romagna Hub Laboratory (20 units), according to the Italian law [17]. To perform the study, selected donors were phenotypically identical in couples (AB0, Rh and Kell identical) and their whole blood units were pooled together before the preparation of P-RBCs with the two methods in order to exclude donors’ variability.

The main characteristics of blood donors recruited in the study are summarized in the Results section and in Supplementary Table S1.

### 2.3. Packed RBCs and Samples Preparation

**Figure 1:**
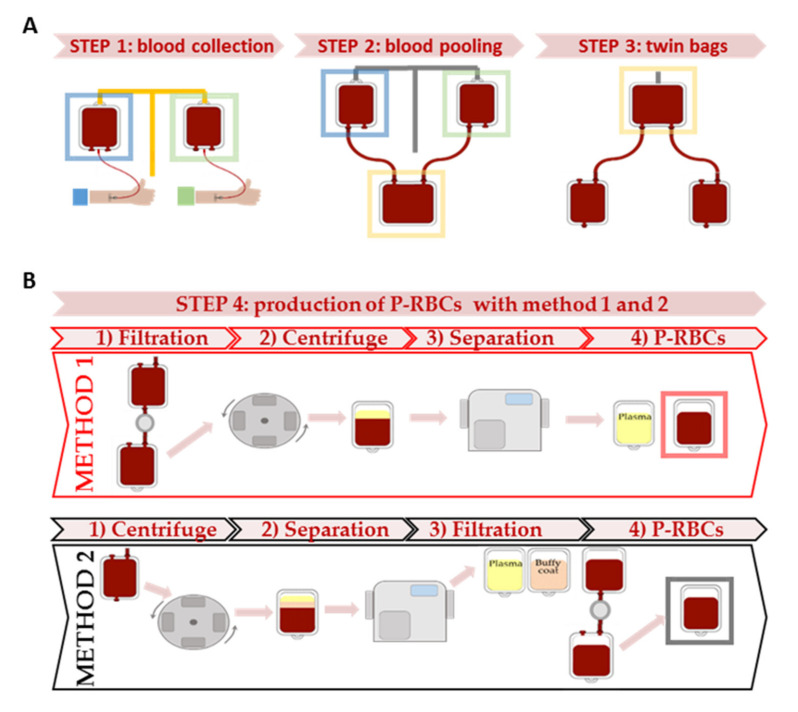
Scheme of experimental procedures. (**A**) in step 1 blood units are collected at day 0 from couples of donors with identical blood groups phenotype. Blood donors enrolled were 40, 20 from two different centers. In step 2, blood units from a couple of matched donors are pooled together in a transfer bag. In step 3, whole blood in the transfer bag was separated in 2 twin satellite bags. (**B**) in step 4 P-RBCs (red or black square) are prepared with method 1 or 2. The initial blood units are the two identical twin bags prepared in step 3.

Blood units were collected in CPD (Citrate, Phosphate and Dextrose) anticoagulant solution. Two units from donors with identical phenotype were pooled together (Transfer bag BB*T200BM, Terumo, Tokyo, Japan) and divided in two satellite bags connected to two different in-line blood bag systems (CQ31555, Fresenius HemoCare Italia s.r.l., Mirandola, Italy and CQ32255, Fresenius HemoCare Italia s.r.l., Mirandola, Italy) and a MacoPress Smart EVO (Macopharma, Rho, Italy) in the Romagna Hub Laboratory. The characteristics of whole blood pools are summarized Supplementary Table S2. In method 1, pooled blood units were initially filtered for leucodepletion, centrifuged and separated to obtain pre-storage P-RBCs without plasma in saline-adenine-glucose-mannitol (SAG-M, Fresnius HemoCare Italia s.r.l., Mirandola, Italy) preservative solution, using Fresenius bag CQ31555 (Fresenius HemoCare Italia s.r.l., Mirandola, Italy). In method 2, pooled blood units were centrifuged and separated to remove plasma and buffy coat, using Fresenius bag CQ32255 (Fresenius HemoCare Italia s.r.l., Mirandola, Italy). Subsequently, the P-RBCs units in SAG-M were filtered for leucodepletion. A scheme of the two methods is represented in Figure 1.

In both methods, plasma and buffy coat were removed using a Compomat G4 separator (Fresenius Kabi Medicare, Isola della Scala (VR), Italy). Each final RBCs concentrate was further divided in four satellite bags and stored up to 14 days at 4 °C. Analyses were performed at 2, 7 and 14 days of storage.

Supernatants volumes were obtained by centrifuging P-RBCs units at 2500× *g* for 5 min. For hematology, flow cytometry, hemolysis and erythrocyte fragility assays, 2 mL aliquots of P-RBCs were directly analyzed. For electrolytes, iron metabolism and lactate dehydrogenase (LDH) analyses, 3 mL samples were centrifuged at 3000× *g* for 10 min at 4 °C in Separgel^®^ tubes to obtain supernatants (Elle Plast s.r.l., Milano, Italy). For metabolomics and cytokines analysis, supernatants from P-RBCs were obtained after centrifuge at 3000× *g* for 10 min at 4 °C in sterile plastic tubes (Thermo Fisher Scientific, Waltham, MA, USA). Protease inhibitor cocktail (Sigma-Aldrich, St. Louis, MI, USA) was added to the supernatants destined to cytokines analysis and stored at −20 °C. Supernatants for metabolomics analysis were further processed as described in paragraph 2.8.

### 2.4. Volumes and Protein Content

Total volumes were calculated weighting the P-RBCs units. Supernatants volumes were calculated correcting the total volumes for the corresponding HCT. Total protein content was measured in P-RBCs supernatants using Bio-Rad protein assay dye and according to manufacture instruction (#5000006, Bio-Rad Laboratories, Hercules, CA, USA).

### 2.5. Hematology Analysis

Complete blood count (leucocytes, RBCs, hematocrit (HCT), Hb, mean corpuscular volume (MCV), mean corpuscular hemoglobin (MCH), mean corpuscular hemoglobin concentrations (MCHC), red blood cell distribution width (RDW), mean platelet volume (MPV) and platelets) were measured on 1 mL samples from donors’ whole blood or P-RBCs using a CELL-DYN Ruby Hematology Analyzer (Abbott Laboratories, Chicago, IL, USA).

### 2.6. Leukocytes Quantification and Viability

P-RBCs samples were prepared as previously described [18]. Briefly, residual leucocytes were stained with mouse monoclonal anti-CD45 FITC-conjugated antibody (Beckman Coulter, Brea, CA, USA), while 7-amino actinomycin D (7-AAD, Beckman Coulter, Brea, CA, USA) was exploited to assess their viability. Samples were analyzed by means of flow cytometry (FC 500 flow cytometer, Beckman Coulter, Brea, CA, USA).

### 2.7. Haemolysis and Erythrocytes Fragility

P-RBCs hemolysis was estimated following the Harboe direct spectrophotometric method. Free hemoglobin (HbO_2_) absorbance was measured at 415 nm (extinction coefficient ε = 512 mM^−1^ cm^−1^); the percentage of hemolysis was derived as previously described, using the free hemoglobin concentration in the supernatants, the total hemoglobin and the hematocrit [19]. Erythrocytes fragility was determined in duplicates as follows [20]: 5 μL of P-RBCs were mixed with 95 μL of NaCl solution at decreasing osmolarity: from 300 mOsm (corresponding to 0.9% NaCl) to 0 mOsm (corresponding to pure water), then samples were centrifuged at 2500× *g* for 1 min. Hemoglobin absorbance in the supernatants was measured at 560 nm in a 96-well plate (GloMax plate reader, Promega Corp., Madison, WI, USA). Results were plotted and fitted using a sigmoidal function as previously described [20]. The mean erythrocyte fragility (X_50_) was obtained for each condition.

### 2.8. Cytokines Quantification

For each P-RBCs-derived supernatant sample, cytokines were quantified using the Human Discovery Th1/Th2 Fixed Panel of the Luminex multiplex assay (R&D Systems, Minneapolis, MN, USA) and read with a Bio-Plex^®^ MAGPIX™ Multiplex Reader (Bio-Rad Laboratories, Hercules, CA, USA). IL-1β, IL-2, IL-4, IL-5, IL-6, IL-10, IL-12, IL-13, GM-CSF, TNF-α, and IFN-γ analyses were performed in triplicate following the manufacturer’s instruction.

### 2.9. Electrolytes, Iron Metabolism and LDH Analysis

LDH, Cl^-^, Na^+^, K^+^, Mg^2+^, NH_4_^+^, iron, transferrin, total iron binding capacity (TIBC), latent iron binding capacity (LIBC) were measured with an Atellica CH 930 Analyzer (Siemens Healthineers AG, Erlangen, Germany) on P-RBCs supernatants following manufacturer’s instruction. Ferritin was quantified on supernatants using an Architect (Abbot Laboratories, Chicago, IL, USA).

### 2.10. Proton Nuclear Magnetic Resonance (^1^H-NMR) Metabolomics Analysis

Samples (2 mL) collected from P-RBCs supernatants were filtered by centrifugation (3000× *g* for 10 min) on Amicon Ultra-4 Centrifugal filters (3000 MWCO, Merck Millipore, Burlington, MA, USA) to remove proteins and lipids, and stored at −20 °C before analysis. For metabolomics, 570 μL of filtered sample was mixed with 30 μL of 1% 3-trimethylsilyl propanoic acid (TSP, as chemical shift reference and quantitative internal standard) in D_2_O and 10 μL of 1 M phosphate buffer (Sigma-Aldrich, St. Louis, MI, USA) to adjust the pH (7.4). High-resolution one-dimensional (1D) ^1^H-NMR spectra were acquired on a JEOL 600 MHz ECZ600R spectrometer using the first increment of NOESY pulse sequence, 15,000 Hz spectral width, 128 scans, 64 k points, 5 s relaxation delay, at 25 °C. The spectra were processed and analyzed with Chenomx NMR suite 8.3 (Chenomx Inc., Edmonton, AB, Canada), zero-filing to 256 k points and line broadening 0.5 Hz.

### 2.11. Statistical Analysis

Data are expressed as mean ± standard deviation (SD) or standard error of the mean values (SEM). Unpaired *t*-tests were carried out for statistical comparisons between the two methods. Differences with a *p* ≤ 0.05 were considered statistically significant. Data analyses were developed on GraphPad Prism 8.4.2 (GraphPad Software Inc., CA, USA) and Excel 2010 (Microsoft, Redmond, WA, USA).

## 3. Results

### 3.1. Study Population

In this study, we enrolled 40 male donors (38.7 ± 13.9 years old), with mean Hb of 15.5 ± 0.9 g/dL and mean HCT of 45.3 ± 2.5 %. Details are shown on Supplementary Table S1. Donors were selected in phenotypically identical couples, from whom we obtained 20 pooled whole blood units (Figure 1A), with 13.5 ± 0.6 g/dL Hb and 40.0 ± 2.1 % HCT. Collected whole blood is diluted due to the CPD anticoagulant contained in the collection bags: therefore, mean Hb concentration and other blood parameters of pooled units were lower than the values measured in donors before collection. Detailed hematological, phenotypical and biochemical features of all the pooled blood units considered are summarized in Supplementary Table S2.

Subsequently, P-RBCs were obtained following two different methods of preparation (Figure 1B) and characterized for various parameters.

### 3.2. Characteristics of the P-RBCs Units and RBCs Indices

P-RBCs volumes and total protein content (both measured at day 2 after donation) were significantly different in the two methods (Table 1). pH were significantly different at all the time points (Figure S1), while P-RBCs supernatants volume, although slightly variable, were did not differ between the two methods (Table 1). Of note, we observed that P-RBCs volume was higher for method 1: this difference might be partially attributable to the buffy-coats separation step (Figure 1), which is performed only for method 2 (the mean volume of buffy-coats collected from method 2 was indeed 56 ± 5.8 mL). Hb, RBCs, HCT and WBCs were normalized for the unit volumes, while other concentration parameters were normalized for the supernatant volumes.

**Figure 2:**
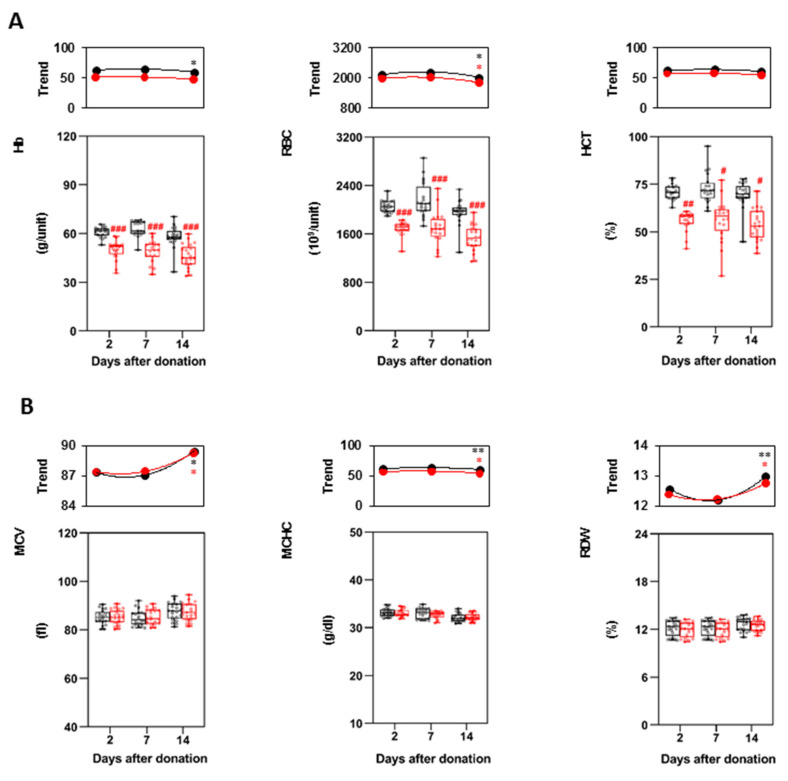
Box plots with single values of RBCs indices at 2, 7 and 14 days of storage. (**A**) Hb, RBCs, HCT values; (**B**) MCV, MCHC and RDW. Method 1 is in black, method 2 in red. The boxplots represent the interquartile range, while the internal line indicates the median; the whiskers span from minimum to maximum. *N* = 20 single samples are represented for each condition. Points and lines in the top panel indicated means of the box plot values and their trend lines during storage, asterisks highlight significant differences between two consecutive time points Red # indicate significant differences between the two methods at the different time points, Black * indicate significant differences of the same method at two consecutive time points. # and * *p* < 0.05, ## and ** *p* < 0.01, ### *p* < 0.001.

Hb, RBCs and HCT were all found to be significantly lower in method 2 at all the time points. Their count remained stable between day 2 and 7, while RBCs decreased at day 14 in both methods and Hb only in method 1 (Figure 2A). Hb, RBCs and HCT values between the two methods were estimated to differ of around 20% at all the time points. Subtracting to this comparison the number of RBCs measured in the buffy-coats units (238 ± 65 × 10^9^ total RBCs/unit), the difference in RBCs concentration decreased to around 10%, suggesting that part of the RBCs in method 2 (which are leucodepleted when already packed) are probably lost during filtration. Conversely, RBCs indices (MCV, MCHC, and RDW) did not differ in the P-RBCs obtained following the two methods, albeit MCHC trends resemble those of Hb, RBCs and HCT. MCV and RDW have instead an inverse trend, slightly increasing from day 7 to day 14 (Figure 2B), thus indicating RBCs aging. These values were not substantially different before and after leucodepletion (Table S2), suggesting that filtration did not alter erythrocytes features.

### 3.3. WBCs and PLTs

**Figure 3:**
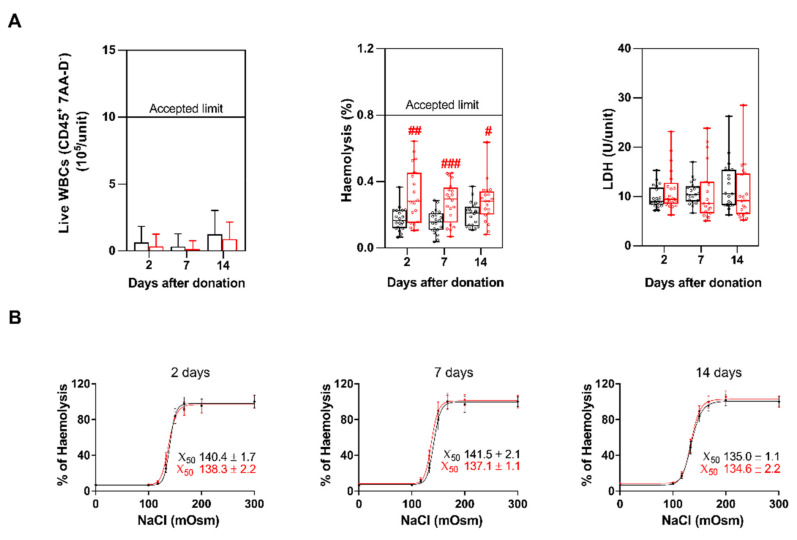
Residual live WBCs, hemolysis, LDH and erythrocytes fragility in the two methods at 2, 7, and 14 days after storage. (**A**) Mean live WBCs ± SD, single values of the percentage of hemolysis, and single values of LDH levels. *N* = 20 samples were collected for each condition; (**B**) Erythrocytes fragility at all the time points. Each value was collected in triplicate for *n* = 20 samples and expressed as mean ± SD. Points were fitted using a sigmoidal function to obtain the X_50_ values reported in the figures. Method 1 is in black, method 2 in red. The boxplots were prepared as described in Figure 2. Red # indicate significant differences between the two methods at the various time points. # *p* < 0.05, ## *p* < 0.01, ### *p* < 0.001.

Live WBCs (CD45^+^/7AA-D^−^) were assessed in P-RBCs samples by flow cytometry. Their mean concentration was always far below the limit of 1 × 10^6^ cells/unit in both methods, and the mean count was included in the optimal range (0.5–1 × 10^5^ cells/unit) [9,21] as indicated by the Italian law [17] (Figure 3A). Residual PLTs were less than 3% of the initial concentration, with a significantly lower concentration in P-RBCs prepared with method 2 (Figure S1).

### 3.4. Haemolysis, LDH and Erythrocytes Fragility

Hemolysis was tested by spectrophotometrically measuring the absorbance of free Hb. The % of hemolysis was always below the accepted limit of 0.8% [19]; nevertheless, in method 2 we found significant higher values of hemolysis at all time points compared to method 1 (Figure 3B, first panel).

LDH levels were previously correlated with hemolysis and proved to increase during storage [22]. Herein we did not find a significant difference in the LDH levels between the two methods, although we measured a significant increase of LDH from day 2 to day 7 in method 1 (Figure 3B, second panel).

Conversely, the estimated erythrocytes mean fragility (X_50_) was slightly lower in method 1 up to day 7, while it was almost identical at day 14, due to an increased fragility of erythrocytes prepared with method 1 at this time point (Figure 3B).

### 3.5. Cytokines Content

Cytokines were measured on P-RBCs supernatants by means of ELISA assay at all the time points. The concentrations of cytokines were low, included in the lower limit of detection or below it. Significant higher values of IL-4 (only after 2 days) and 10 (only after 14 days) were measured in method 1, respectively with a fold change of 1.9 and 2.2 (Table 2).

### 3.6. Iron Metabolism

**Figure 4:**
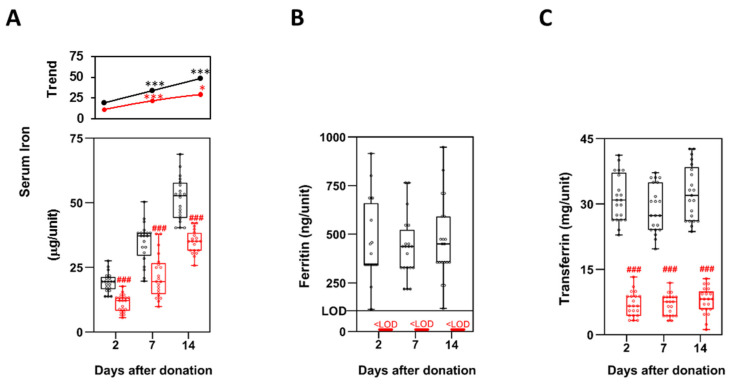
Iron metabolism in the two methods at 2, 7 and 14 days after storage, expressed as concentration/unit. In serum iron (**A**), points and lines in the top panels of (**A**) indicate differences between consecutive time points, as described in Figure 2; In the Ferritin (**B**) and Transferrin (**C**) panels, the horizontal line indicates the limit of detection: values measured in method 2 samples were all below this limit (1 ng/mL according to manufacturer’s, which here is normalized for the maximum unit volume measured, 180 mL). Method 1 is in black, method 2 in red. The boxplots were prepared as described in Figure 2. Red # indicate significant differences between the two methods at the various time points. ### *p* < 0.001. * *p* < 0.05, *** *p* < 0.001.

As already observed for Hb (Figure 2A), serum iron levels were also significantly higher in P-RBCs prepared with method 1 at all the time points considered, with values almost always twice than in method 2 (Figure 4A). Iron release in the supernatant increased during storage: as highlighted by the trend lines (Figure 4A, upper panel), the difference in iron concentration between 2 and 14 days was significantly higher for method 1 (29 μg/unit) than for method 2 (18 μg/unit).

Ferritin concentration was also found to be over four times higher in method 1. Nevertheless, its concentration in method 2 was below the limit of detection and it was not possible to determine the statistical significance (Figure 4B). Similarly, transferrin concentrations (mg/unit) were significantly higher in method 1, with a fold change between 4.2 and 5.0 (Figure 4C). Both ferritin and transferrin concentrations did not change during storage time.

### 3.7. Metabolites and Electrolytes

**Figure 5:**
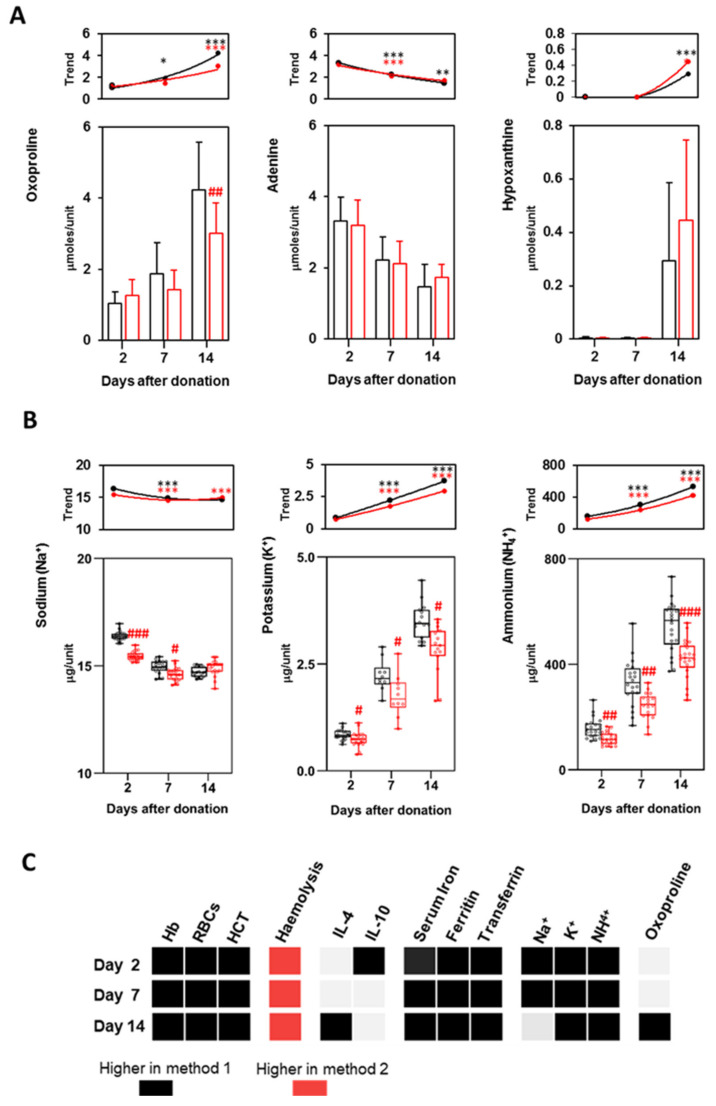
Metabolites and electrolytes comparison at 2, 7 and 14 days after storage. (**A**) Bars of the concentration/unit of oxoproline, adenine and hypoxanthine; (**B**) Box plots with single values of the concentration/unit of K^+^ and NH_4_^+^. Points and lines in the top panels of both (**A**,**B**) indicate differences between consecutive time points, as described in Figure 2. Method 1 is in black, method 2 in red. The boxplots were prepared as described in Figure 2. Red # indicate significant differences between the two methods at the various time points. # *p* < 0.05, ## *p* < 0.01 and ### *p* < 0.001. * indicate significant differences of the same method at two consecutive time points. * *p* < 0.05, ** *p* < 0.01 and *** *p* < 0.001; (**C**) Summary scheme of the significant different parameters between the two methods at all the time points considered.

Main metabolites, proposed as biomarkers of metabolic lesions, were measured by means of ^1^H-NMR [23]. Oxoproline, a marker of oxidative stress, [11,23] significantly increased over storage time with both methods, and it was significantly higher in method 1 than in 2 at 14 days. Conversely, adenine was metabolized over time as previously reported [11]: although units prepared with method 1 contained a higher number of RBCs, it was not observed a significantly higher consumption of adenine at any time point. Hypoxanthine was almost absent until 14 days, when its levels increased in both methods (Figure 5A). The variation of these metabolites over time, together with the acidification of the supernatant (Figure S1B), are in accordance with what previously reported and indicate the appearance of lesions in stored RBCs [23]. Nevertheless, in our study only oxoproline differentiated the two methods, and only at 14 days.

Increasing levels of Na^+^, K^+^ and NH^4+^ (Figure 5B), together with the decrease in Cl^−^ concentrations (Figure S1C), were proved to be evidence of storage lesions [10,23]. Herein, electrolytes were all found to be significantly lower in method 2 at all the time points considered. Na^+^ concentration slightly decreased between 7 and 14 days of storage. On the contrary, K^+^ and NH^4+^ significantly increased during the storage of P-RBCs prepared with both methods: trend lines highlight a faster increase at 14 days for these latter metabolites in method 1 (Figure 5B). Little but significant lower concentrations of Mg^2+^ were measured in method 2, its level remained constant over storage (Figure S1C).

## 4. Discussion

For βTM patients, RBC transfusion is a lifelong and frequent treatment that severely affects quality of life. Different manufacturing protocols can influence P-RBCs quality, thus affecting both the Hb need and the risk to develop possible complications in the recipient. The Thalassemia International Federation recently reported that filtration of whole blood is “the preferred method for leuco-reduction’’ of P-RBCs [24], since it suggests that this method may effectively reduce leucocytes to a minimal level, ensuring at the same time a high RBCs (and Hb) recovery. Nevertheless, both method 1 and 2 are accepted and routinely used to prepare P-RBCs for βTM blood therapy [9] and up to 2021 there were not reported studies comparing leucodepleted P-RBCs obtained with alternative procedures from whole blood.

In a recent one-year crossover clinical trial [16], the procedure suggested by the Thalassemia International Federation (here method 1) was compared with another method routinely used to prepare P-RBCs for βTM patients [9], where whole blood is first separated to obtain packed erythrocytes, which are subsequently filtered (here method 2). Method 1 was proved to produce P-RBCs with a higher content of Hb, which in turn resulted in a longer transfusion interval (14.8 vs. 14.2 mean days between two consecutive transfusion episodes). Despite the trial by Gamberini and colleagues [16] has the merit to be the first attempt to find the most suitable product for βTM patients transfusion, several important aspects remain to be elucidated to identify the most effective and safest P-RBCs product.

Particular attention, in this context, should be given to markers that may influence iron overload, such as proteins involved in its metabolism. Since βTM patients often receive transfusions for their whole life, RBCs quality should be as important as Hb content. In recent decades, indeed, research on blood components highlighted the importance of storage lesions for the proper activity of transfused RBCs [9,10,11,12,22,23].

Our study compares the two procedures over time, evaluating the leucodepletion efficiency as well as the erythrocytes integrity, the supernatant composition and consequences of the method of preparation on the development of storage lesions and release of inflammation mediators. Here we provide an in-depth characterization of the biochemical properties of P-RBCs prepared from pooled whole blood. This approach allowed us to minimize the inter-individual variability.

In accordance with previously reported results [16], we measured a higher Hb content for P-RBCs prepared with method 1 (18–20% more than method 2). The RBCs count (18–23% more than method 2) and HCT levels increased accordingly. In our in vitro study we could not quantify the impact of this difference on post-transfusion blood viscosity nor to infer if a higher HCT level might exert a positive or negative effect: nevertheless, literature data on βTM patients report a range of 55–60% as optimal values for HCT levels in red cell concentrates [25], thus suggesting that both methods are adequate.

P-RBCs prepared with method 1 had lower hemolysis, although this parameter was largely below the accepted limit (0.8%) in both procedures and at all the time points. Other values correlated with RBCs rupture (i.e., LDH release and erythrocytes fragility) were not statistically different. Since in method 2 RBCs are filtered when already packed, the higher hemolysis might be attributed to an initial damage of RBCs during filtration, as previously reported, [14] which however did not alter erythrocytes over time. In accordance with this hypothesis, we did not find differences in RDW trends between the two methods. Small changes in MCV and MHCH were observed at 14 days, confirming that RBCs changes occur during storage [26], albeit not correlated with the method of preparation.

Since different leucodepletion methods affect both the efficacy and the quality of P-RBCs [14,15], here we checked for residual leucocytes and pro-inflammatory mediators that eliciting adverse immune response, may produce negative effects on transfused patients [27]. Despite remaining always below the legal limits, WBCs concentrations were slightly higher in method 1. Pro-inflammatory cytokines were always low or even undetectable, and their levels were comparable to what was previously published for P-RBCs [28,29]. Anti-inflammatory cytokines IL-4 and 10 were higher in P-RBCs prepared with method 1, although their concentration should not be sufficient to affect the immune status of the recipient [30,31].

A vast scientific literature reports that biochemical, structural, and metabolic features of RBCs (i.e., decreased RBCs recovery, impaired oxygen transport and development of potentially toxic derivatives) are affected by the storage time [10,23,32,33,34]. Although major storage lesions occur two weeks after blood collection, several metabolic changes already appear between 7 and 14 days [10,23,32,33,34], which is the maximum allowed storage time for transfusion products destined to βTM patients. The possible negative consequences of these by-products of RBCs senescence and damage are amplified in multi-transfused individuals. In the supernatants of P-RBCs prepared with method 1 we observed a significantly higher accumulation of oxoproline, which is correlated with GSH turnover and oxidative stress [32,33]. The latter is mainly mediated by Fenton reactions involving ferric iron in hemichromes, free haem and molecular iron released from haem [35], which are all by-products of Hb. In P-RBCs prepared with method 1 we found a higher concentration of both Hb and iron, which in turn may result in the development of reactive oxygen species (ROS) with a higher rate. Conversely, adenine consumption and the consequent hypoxanthine production did not significantly differ between the two methods, despite the higher content of RBCs in method 1, possibly because of the large excess of adenine in the storage medium. Supernatant pH variations suggest that also lactate has been produced at the same rate, and that energy metabolism did not significantly differ in both methods. In a recent in vitro study, we reported that high hypoxanthine levels in stored P-RBCs units may be responsible for neutrophils activation [36]: however, in both P-RBCs procedures herein tested hypoxanthine levels were below the critical value.

The electrolytes measured in the supernatants confirmed that RBCs develop more storage lesions in method 1. NH_4_^+^, Cl^−^, Na^+^ and K^+^ fluctuations were previously proved to be evidence of these processes [10,23,27,32]. Potassium, in particular, accumulates in the supernatants as a consequence of the impaired function of the Na^+^/K^+^ pump [27,32,37], and its normalization in transfused patients may take up to 4 days [27]. Due to this delay in K^+^ normalization, massive transfusions are known to cause cardiac arrest on infants and new-borns [38]; in addition, 40 days-old blood products (when K^+^ was less than twice more concentrated than at 7 days) altered the cardiac electrophysiology in in vitro experiments [37]. Although there is no clear evidence correlating K^+^ levels and post-transfusion complication, this aspect should be further investigated in βTM patients, which are multi-transfused and commonly affected by iron overload-related cardiac problems.

Among the ions found in stored P-RBCs, iron holds a position of primary importance, being its overload the main cause of death for βTM patients. Iron concentration in P-RBCs supernatants raises during storage [10], and transfusion with old P-RBCs causes an increase of non-transferrin-bound iron and the related iron toxicity [39]. We showed that iron concentration is significantly higher in P-RBCs prepared with method 1 already two days after blood collection; albeit accumulating in the supernatants over storage in both methods, its fold change (1.6–1.7) remained stable at the different time points. Supernatant ferritin and transferrin were also found to be higher, despite their concentrations reflect the higher amount of supernatant proteins measured in method 1. In their recent crossover study on βTM patients alternatively transfused with the two types of P-RBCs, Gamberini and colleagues [16] assessed ferritin concentration in patients only over a six months period, without considering any other aspect of iron overload. It would be indeed of fundamental importance to assess whether frequent transfusions with method 1 P-RBCs might negatively impact on iron overload over a longer period of time (years).

## 5. Conclusions

Altogether, our results demonstrate that different methods for producing P-RBCs may significantly impact on their biochemical features. Hb and RBCs concentrations, as well as an efficient leucodepletion, are of fundamental importance for the transfusion therapy of βTM patients: nevertheless, we showed that other parameters, such as iron, potassium concentrations and oxidative stress markers have to be considered in order to select the best procedure. Clinical studies taking into account the possible long-term consequences of these aspects on βTM patients are necessary to find a consensus on the methodological procedure that may assure to obtain the best product for this purpose.

## Figures and Tables

**Table 1:** Total volumes, total protein content and supernatant volumes of the P-RBCs prepared with the two methods.

	Method 1	Method 2	*p*-Value
**Total volume (mL/unit)**	303 ± 12	258 ± 14	<0.01
**Total protein (g/unit)**	12.3 ± 2.6	7.2 ± 1.9	<0.05
**Supernatant volume (mL/unit)**			
**2 days**	114 ± 10	109 ± 13	n.s.
**7 days**	108 ± 22	110 ± 29	n.s.
**14 days**	118 ± 18	115 ± 21	n.s.

n.s.: non-significant.

**Table 2:** Cytokine levels in the two methods at the different time points. *p*-value is reported when significant.

Storage	Method	IFN-γ	IL-1β	IL-2	IL-4	IL-5	IL-6	IL-10	TNF-α
**2 days**	**1**	502 ± 308	176 ± 51	143 ± 100	120 ± 103	187 ± 49	303 ± 97	6.8 ± 2.4	155 ± 130
	**2**	447 ± 202	97 ± 36	98 ± 32	166 ± 106	232 ± 12	545 ± 19	3.5 ± 1.8	68 ± 48
								*p* < 0.05	
**7 days**	**1**	314 ± 127	96 ± 36	61 ± 36	148 ± 106	154 ± 12	204 ± 19	4.4 ± 1.8	74 ± 48
	**2**	448 ± 215	90 ± 44	91 ± 42	76 ± 48	145 ± 18	224 ± 40	4.8 ± 2.6	81 ± 54
**14 days**	**1**	296 ± 202	92 ± 160	90 ± 150	136 ± 60	193 ± 86	298 ± 146	3.3 ± 1.3	96 ± 53
	**2**	422 ± 252	133 ± 11	85 ± 42	62 ± 31	145 ± 7	218 ± 22	3.5 ± 2.8	52 ± 24
					*p* < 0.05				

GM-CSF, IL-12 and 13 are not included in the table as they were below the detection limits, respectively of 1.2, 2 and 3 pg/unit.

## Data Availability

The data presented in this study are available on request from the corresponding author. The data are not publicly available due to privacy and ethical restrictions.

## Supplementary Materials

The following are available online at https://www.mdpi.com/article/10.3390/biom11111638/s1, Supplementary Figure S1: Platelets count, pH and electrolytes comparison. Supplementary Table S1: Main features of donors’ whole blood before collection. Supplementary Table S2: General features of the pooled whole blood before processing.
