# Supplementary material for: Comparison of Two Alternative Procedures to Obtain Packed Red Blood Cells for β-Thalassemia Major Transfusion Therapy
Source: Biomolecules. 2021 Nov 4;11(11):1638. doi: 10.3390/biom11111638 (PMC8615631; doi:10.3390/biom11111638)
Supplement: Supplementary file 1 [file biomolecules-11-01638-s001.zip › biomolecules-1400727-supplementary.pdf]

biomolecules

MDPI

Supplementary Information

1. Supplementary Figures

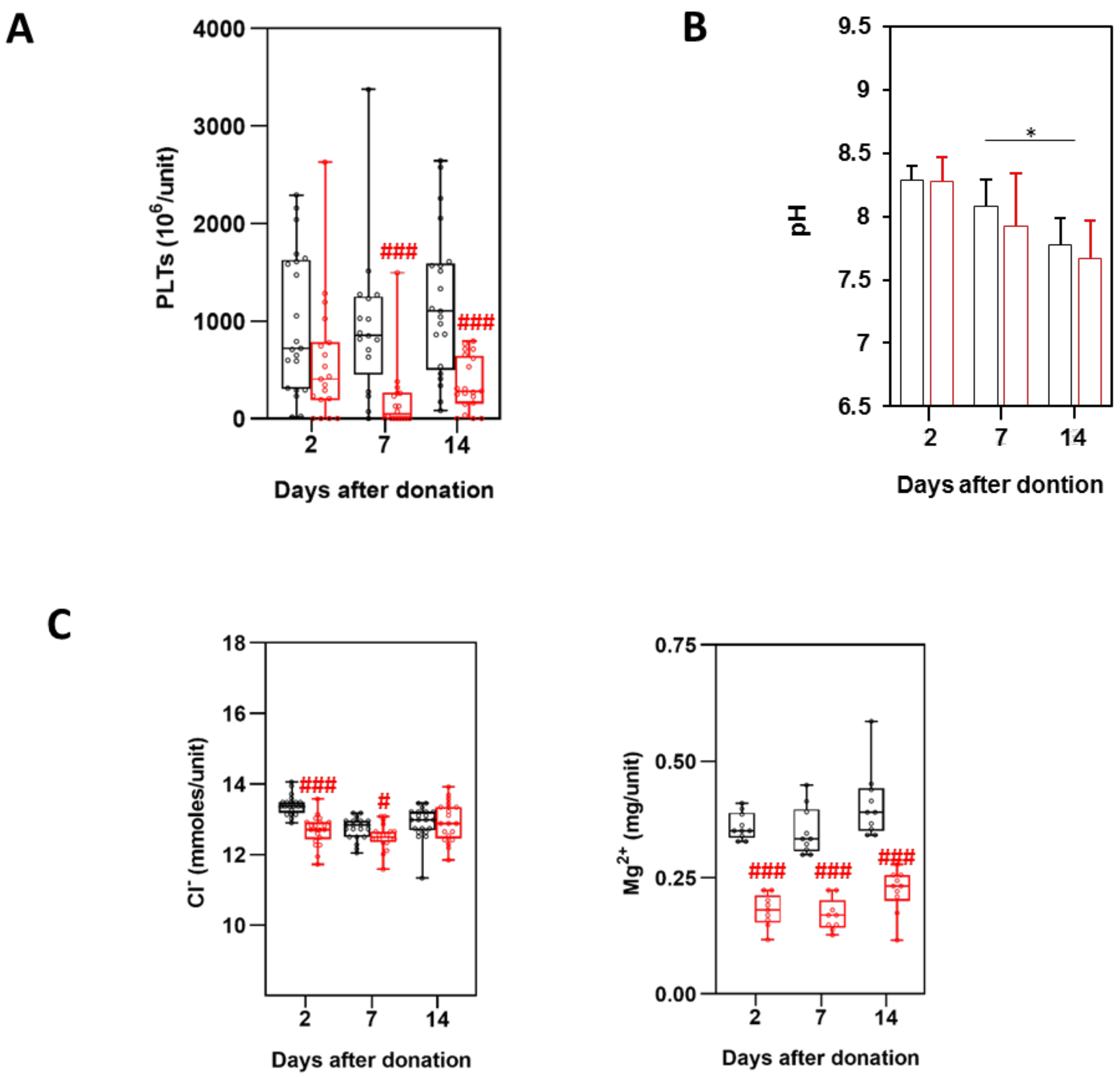

Figure S1. Platelets count, pH and electrolytes comparison. (**A**) Box plots with single values of the number of platelets/unit at 2, 7 and 14 days after storage; (**B**) Bars of the pH values in the supernatants. Between 12 and 15 were analyzed for each condition; (**C**) Box plots with single values of the concentration/unit of Cl^- and Mg^{2+}. Method 1 is in black, method 2 in red. The boxplots were prepared as described in Figure 2. Red # indicate significant differences between the two methods at the various time points. # $p <$ 0.05 and ###.

2. Supplementary Tables

Table S1. Main features of donors' whole blood before collection.

Donor	RBCs (× 10^{12}/L)	Hb (g/dL)	HCT (%)
1	4.7	14.0	42.8
2	5.5	16.0	47.3
3	5.3	16.1	47.6
4	4.9	14.7	42.1
5	4.7	15.1	44.5
6	5.0	14.6	42.4
7	5.4	15.5	45.5
8	5.0	14.7	42.9
9	4.7	14.8	41.9
10	5.1	15.1	43.7
11	5.3	15.2	45.0
12	5.2	15.3	45.4
13	5.0	16.3	47.1
14	5.3	15.4	43.8
15	4.9	14.2	41.2
16	4.9	15.3	43.6
17	5.0	14.6	42.8
18	5.1	14.5	42.2
19	5.2	13.6	41.0
20	5.3	15.8	44.8
21	5.3	16.8	46.9
22	5.0	14.8	44.7
23	5.3	15.2	44.4
24	5.5	15.0	45.1
25	5.4	16.0	47.5
26	5.4	16.7	46.6
27	5.4	15.6	46.7
28	5.5	16.2	47.3
29	5.0	14.9	44.6
30	6.2	16.1	50.7
31	5.3	16.5	45.5
32	5.4	15.3	46.3
33	5.4	15.9	46.5
34	5.3	15.5	45.4
35	5.4	15.7	46.8
36	5.7	17.5	50.7
37	5.9	17.1	49.7
38	5.8	17.6	50.4
39	5.3	15.5	45.3
40	5.4	15.5	45.0

Table S2. General features of the pooled whole blood before processing.

n	Phenotype	WBC (10^3/μl)	RBCs (10^6/μl)	Hb (g/dl)	HCT (%)	MCV (fl)	MCH (pg)	MCHC (g/dl)	RDW (%)	PLTs (10^3/μl)	MPV (fl)	Ferritin (ng/ml)	Serum Iron (μg/dl)	TIBC (%)	LIBC (%)	Transferrin saturation (%)	Transferrin (mg/dl)	Na^+ (mM)	K^+ (mM)	Cl^- (mM)	Mg^{2+} (mg/dl)	NH^{4+} (μg/dl)	LDH (U/l)
1	A+ CcDeekk	4.08	4.47	13.9	39.2	87.8	31	35.4	11.7	92.4	6.4	46	63	238.7	175.7	26.3	188	166.7	3.5	78	1.55	132	346
2	A+ CcDEekk	5.25	4.42	13.4	39.5	89.4	30.4	34	13.1	162	7.95	25	125	274.3	149.3	45.5	216	167	4.7	79	1.56	116	291
3	0+ CcDeekk	3.41	4.13	13	37.4	90.7	31.4	34.6	11.4	148	7.64	41	67	264.1	197.1	25.3	208	166.6	4.9	78	1.54	132	279
4	A+ CcDeekk	4.16	4.46	13.1	38.2	85.7	29.3	34.2	10.7	139	5.24	35	96	276.8	180.8	34.6	218	168.8	3.3	78	1.53	103	335
5	AB + CcDeekk	4.36	4.33	13.3	38	87.9	30.7	34.9	10.5	111	6.99	20	65	242.5	177.5	26.8	191	164.4	5.3	77	1.52	137	340
6	0 + CcDeekk	5.1	4.52	13.2	38.8	85.8	29.2	34	10.6	161	7.11	45	56	241.3	185.3	23.2	190	168.9	4.1	79	1.53	114	325
7	0 + CCDeekk	7.6	4.66	13.8	40.8	87.5	29.5	33.7	12.4	184	8.33	123	86	259	173	33.2	204	168	3.7	77	1.57	122	288
8	B + CcDeekk	4.97	4.23	13.1	38.1	90.1	30.9	34.3	13.1	108	8.91	31	88	275.5	187.5	31.9	217	167.3	3.5	79	1.58	98	359
9	A+ CcDeekk	3.38	5.1	14.8	45.1	88.6	29	32.7	13.1	136	8.03	80	47	246.3	199.3	19	194	166.6	3.4	80	1.67	125	309
10	A+ CCDeekk	5.14	4.82	13.5	41.5	86.2	28.1	32.6	13.6	137	8.05	72	77	242.5	165.5	31.7	191	168.3	3.3	78	1.64	104	296
11	A+ CcDeekk	5.25	4.36	12.8	38.5	88.3	29.4	33.2	13.6	197	9.6	22	93	298.4	205.4	31.1	235	156	3.9	81	1.59	190	211
12	A+ CcDeekk	6.24	4.65	13.3	40.6	87.3	28.6	32.8	13.7	170	10.6	28	75	288.2	213.2	26	227	157	4.3	80	1.7	222	245
13	A+ CcDeekk	4.38	4.48	13.4	40.1	89.5	29.9	33.4	13.2	164	10.9	22	68	304.8	236.8	22.3	240	158	4.3	83	1.69	167	163
14	A+ CcDeekk	4.11	4.65	13	39	85.6	28	32.7	13.7	151	10.2	29	97	280.6	183.6	34.5	221	155	4.5	78	1.55	218	186
15	A+ CcDeekk	4.9	4.43	13.3	39.2	88.5	30	33.9	12.2	124	10.7	58	65	259	194	25	204	156	4.4	81	1.53	215	211
16	A+ CcDeekk	4.69	4.98	14.8	44.7	89.8	29.7	33.1	13.2	103	11	47	107	259	152	41.3	204	157	4.3	81	1.62	197	213
17	A+ CcDeekk	7.39	4.58	13.2	39.7	86.7	28.8	33.2	12.9	191	10.6	44	85	304.8	219.8	27.8	240	157	4.7	84	1.86	279	225
18	A+ CcDeekk	5.31	4.58	13.8	41	89.5	30.1	33.7	12.9	189	10.2	27	90	265.4	175.4	33.9	209	158	4.6	82	1.57	188	156
19	A+ CcDeekk	4.88	4.88	14.1	42.6	87.3	28.9	33.1	12.6	209	10.4	24	75	283.2	208.2	26.4	223	159	4.5	83	1.54	226	197
20	A+ CcDeekk	4.6	4.46	13	38.4	86.1	29.1	33.9	13	173	10.6	31	80	295.9	215.9	27	233	158	4.2	83	1.73	184	158
